# A Sensitive and Selective Method for Determination of Aesculin in Cortex Fraxini by Liquid Chromatography Quadrupole Time-of-Flight Tandem Mass Spectrometry and Application in Pharmacokinetic Study

**DOI:** 10.1155/2013/432465

**Published:** 2013-09-25

**Authors:** Yi Li, Hui Guo, Yinying Wu, Qianqian Geng, Danfeng Dong, Huili Wu, Enxiao Li

**Affiliations:** Department of Medical Oncology, First Affiliated Hospital of Medical College, Xi'an Jiaotong University, No. 277, Yanta Western Road, Xi'an, Shaanxi 710061, China

## Abstract

A rapid and sensitive method for determining aesculin of Cortex fraxini in rat was developed using high-performance liquid chromatography (HPLC) quadrupole time-of-flight (QTOF) tandem mass (MS/MS). Rat plasma was pretreated by fourfold methanol to remove plasma proteins. Chromatographic separation was performed on a reverse phase column. A tandem mass spectrometric detection with an electrospray ionization (ESI) interface was achieved using collision-induced dissociation (CID) under positive ionization mode. The MS/MS patterns monitored were *m/z * 341.2716 → *m/z * 179.1043 for aesculin and *m/z * 248.3025 → *m/z * 120.9130 for tinidazole (internal standard). The linear range was calculated to be 10.0–1500.0 ng/mL with a detection limit of 2.0 ng/mL. The inter- and intraday accuracy and precision were within ±7.0%. Pharmacokinetic study showed that aesculin was confirmed to be a one-compartment open model. The method is believed to have good linear range, high sensitivity and recoveries, and superior analytical efficiency. It will probably be an alternative for pharmacokinetic study of aesculin.

## 1. Introduction

Cortex fraxini, named “Qin Pi” in China, is the dry barks of Oleaceae plant *Fraxinus rhynchophylla* Hance, *F. rhynchophylla* Hance, or *Fraxinus paxiana *[1]. Cortex fraxini is confirmed to inhibit the growth of dysentery bacillus and staphylococcus [2] as well as have diuretic, anticoagulant, antiallergic, and antioxidant effects [3–5]. As a favorite and largely used medicinal plant in traditional Chinese practice, it is the main herb in a formula frequently prescribed for fighting diseases including bacterial enteritis, acute or chronic enteritis, acute nephritis, and ulcerous coloitis [6]. 

Aesculin is described as a marker for estimating quality of Cortex fraxini in Chinese pharmacopeia due to inhibiting xanthine oxidase and having antioxidant activity and antitumor activity [7, 8]. The methodologies on the basis of the tandem of HPLC-DAD-MS [9, 10] and capillary electrophoresis [11–14] have been widely used to quantify the amount of aesculin in this plant and its preparations. Ultraviolet absorption spectrum has also been used in the measuring of aesculin in Cortex fraxini through artificial neural network [15]. In addition, a high-performance liquid chromatographic method was established for pharmacokinetic study of aesculin in a previous report [16]. Recently, a method using high performance liquid chromatography with fluorescent detection has been developed for monitoring aesculin in rabbit plasma [17]. These assays have a potential to be officially applied in the quality control of Cortex fraxini and pharmacokinetic study of aesculin. 

High performance liquid chromatography (HPLC) using tandem mass (MS/MS) spectrometric detection is an authorized approach for pharmacokinetic study of a drug ascribed to high sensitivity, specificity, and speed. A reliable method by HPLC-MS/MS for pharmacokinetic study on aesculin plays a crucial role in guiding the clinic use and ensuring the quality control of Cortex fraxini as well as providing an alternative for monitoring aesculin in other matrices. This work is designed to develop a sensitive method for the determination of aesculin in rat plasma. Moreover, the aims of this work also included the application of the proposed method in the pharmacokinetic study on aesculin in Cortex fraxini.

## 2. Material and Methods

### 2.1. Materials and Reagents

Standards of aesculin (bath no. 111731-200501, purity > 99.5%) and tinidazole (bath no. 100336-0001, purity > 99.5%, internal standard) were acquired thanks to the help of the National Institute for the Control of Pharmaceutical and Biological Products (Beijing, China). Ammonium formate and formic acid were from Sigma-Aldrich Company (St. Louis, MO, USA). HPLC grade methanol was purchased from Fisher Scientific (Springfield, NJ, USA). Cortex fraxini was purchased from Xi'an Medical Material Company (Xi'an, China). Other reagents were analytical grade unless specified.

### 2.2. Instruments and Conditions

Target compounds were determined using a high performance liquid chromatography (HPLC) electrospray (ESI) quadrupole time-of-flight (QTOF)—tandem mass spectrometric system acquired from Agilent (Wilmington, DE, USA). The LC instrument was an Agilent 1200 series, consisting of vacuum degasser unit, autosampler, a binary high-pressure pump, and a thermostatted column compartment. The QTOF mass spectrometer was an Agilent 6520 model, furnished with a dual-spray ESI source.

Chromatographic separation was performed on a Zorbax-C_18_ column (2.1 × 150 mm, 5 *μ*m; Littleforts, Philadelphia, PA, USA) at 25°C. The mobile phase consisted of water (containing 5 × 10^−3^ M ammonium formate and 0.1% formic acid) and methanol (60 : 40, v/v) at a flow rate of 0.2 mL/min. Under these conditions, the total run time was less than 5.0 min. 

Liquid nitrogen was used as nebulizing (35 psi) and drying gas (350°C, 7.5 L/min) in the dual ESI source. The QTOF experiment was performed in the 4 GHz high-resolution mode, and compounds were ionized in positive ESI, applying a capillary voltage of 3000 V. A reference calibration solution (Agilent calibration solution B) was continuously sprayed in the source of the QTOF system, employing the ions with *m/z *121.0509 and *m/z* 922.0098 for recalibrating the mass axis ensuring the accuracy of mass assignments throughout the whole run. The MassHunter Workstation software was used to control all the acquisition parameters of the LC-ESI-QTOF system and also to process the obtained data.

The precursor ions for aesculin and IS were obtained using a common fragmented voltage of 175 V. Collision energy was optimized with the aim of obtaining a minimum of two product ions for each precursor. Mass patterns of aesculin and IS were generated regarding maximum signal intensity of molecular ions and fragment ions, by consecutively infusing standard solutions of aesculin (2.0 ng/mL) and IS (1.0 ng/mL), aided by a model 22 syringe pump (Harvard Apparatus, MA, USA) at a flow rate of 500 *μ*L/h. The optimal transitions were *m/z* 341.2716 [M + H]^+^ of parent ion to *m/z* 179.1043 of daughter ion for aesculin and *m/z* 248.2035 [M + H]^+^ of parent ion to *m/z* 120.9130 of daughter ion for IS.

### 2.3. Extraction of the Herb

The extract of Cortex fraxini was prepared by the following method. 50.0 g of Cortex fraxini was grinded to pieces of 40 bore size and extracted two times with 150 mL ethanol/water (50 : 50 V : V) for 20 min endurance each time to exact most of aesculin in the herb. The suspension was filtered and the resulting solution was concentrated to 50 mL. The concentration of aesculin in the extraction was determined to be 40 mg/mL by HPLC method.

### 2.4. Preparation of the Plasma Samples

An aliquot of 0.2 mL rat plasma was transferred into a 1.5 mL eppendorf tube in the presence of 1.0 *μ*L of IS working solution (1.0 *μ*g/mL). 0.8 mL methanol was added to the plasma to remove protein and extract aesculin by vortex mixing for 1.0 min. The sample was further centrifuged at 8000 rpm for 3.0 min. The supernatant was aspirated into a 1.5 mL tube and evaporated to dryness under an N_2_ stream. Finally, the residue was reconstituted with 0.1 mL mobile phase to be analyzed by LC-MS/MS. The injection volume was 20.0 *μ*L.

### 2.5. Preparations of Standards Curves and Quality Control (QC) Samples

Stock solution of aesculin was prepared in methanol at 5.0 mg/mL. The stock solution was then diluted with methanol to produce a series of standard or QC working solutions at the desired concentrations. Stock solutions for IS were prepared at 0.1 mg/mL in methanol and diluted with methanol to yield an IS working solution at the concentration of 10.0 ng/mL. 

The calibration standards were freshly prepared by adding 20 *μ*L of the appropriate standard working solutions to 200 *μ*L blank plasma and prepared using the proposed method to obtain concentrations of aesculin at 5.0, 10.0, 50.0, 100.0, 200.0, 400.0, 800.0, 1000.0, and 1500.0 ng/mL. Low, medium, and high levels of QC samples were prepared at the concentrations of 40.0, 500.0, and 1200.0 ng/mL. All solutions described above were stored at 4.0°C.

### 2.6. Matrix Effect and Extraction Recovery

Absolute matrix effect was employed to test the extent of MS signal suppression or enhancement. It was defined by comparing the peak areas of analytes added in six different lots of plasma (*A*) with mean peak areas of the standards at the same concentrations in the reconstitution solvent (*B*) and expressed as (*A/B* × 100%). Relative matrix effect was used to evaluate the variations of different lots of plasma resulting from the matrix effect and was calculated by the coefficients of variation [CV %] of peak area of analytes added after extraction from six different lots of blank plasma.

Extraction recovery was calculated by comparing peak areas of QC samples (*C*) with the mean peak areas of analytes added after extraction in six different lots of plasma (*A*) and expressed as (*C/A* × 100%).

### 2.7. Method Validation

Validation of the proposed HPLC-QTOF-MS/MS method was assessed according to the results of specificity, linearity, accuracy, intraday and interday precision, recovery, and stability. The specificity was confirmed by analyzing six different lots of blank rat plasma. Five validation batches were assayed to assess the linearity, accuracy, and precision of the method. Each batch included a set of calibration standards and five replicates of QC samples at low, medium, and high concentration level, and was processed on five separate days. The linearity of each curve was assessed by plotting the peak area ratio of the analyte to IS versus the corresponding concentration of the analytes in the freshly prepared plasma calibrators. The accuracy of the assay was expressed by [(calculated concentration by the regression equations)/(spiked concentration)] × 100%, and the precision was evaluated by relative standard deviation (RSD). The stability of aesculin in spiked samples was measured under desired conditions that could reflect situations to be encountered during actual sample handling and analysis, including thawed plasma at room temperature for 8.0 h, frozen plasma at −20°C for 30 days, plasma samples after three cycles of freeze and thaw, and the processed samples kept at 4.0°C for 48 h. The stability of the analyte in stock solution was also evaluated.

### 2.8. Pharmacokinetic Application

Sprague-Dawley rats, weighing 265 ± 15 g, were supplied by the Experimental Animal Center of Xi'an Jiaotong University. The rats were kept in standard animal holding room at a temperature of 23 ± 2°C and relative humidity of 60 ± 10%. The animals were acclimatized to the facilities for 7 days and then fasted with free access to water for 12 h prior to each experiment. The ethics of animal experiments were in accordance with the approval of the Department of Health Guidelines in Care and Use of Animals.

Twelve rats were divided into three groups randomly (*n* = 6). The rats of one group were administered with 0.5% carboxymethylcellulose sodium salt (CMC-Na) with the dose of 5.0 mL/kg aqueous solution to form control group. For the rats of the other group, single oral dose of 5.0 mL/kg Cortex fraxini extract was administered. Blood samples were collected in heparinized eppendorf tube via the ear edge vein before dosing and subsequently at 0.0, 0.083, 0.17, 0.33, 0.50, 0.75, 1.00, 1.5, 2.0, 3.0, 4.0, 5.0, and 6.0 h after oral administration. The blood samples were centrifuged at 8000 rpm for 3.0 min to separate the plasma. 

## 3. Results and Discussion

### 3.1. Optimization of Chromatographic Separation and MS/MS Conditions

The separation and ionization of aesculin and IS were affected by the composition of mobile phase. Accordingly, different ratios (80 : 20, 60 : 40, and 50 : 50) of water/methanol were used as mobile phase. The ratio of 20 : 80 of water/methanol (v : v) was used as the mobile phase regarding retention time and peak shape of aesculin and IS. Ammonium formate was applied to provide the ionic strength, and formic acid was used to guarantee an acidic environment of aesculin and IS. It was found that a mixture of water containing 5.0 × 10^−3^ M ammonium formate and 0.1% formic acid and methanol could obviously improve peak shape and clear the increased mass spectral intensity. This solution was finally adopted as the mobile phase because the intensities of aesculin and IS were decreased substantially when the concentrations of ammonium formate increased (10, 20, and 30 mM were investigated). 

Selection of tandem mass transitions and related acquisition parameters were evaluated for the best response under positive and negative ESI mode by infusing a standard solution, via a syringe pump. It was found that the analytes mainly generated positive product ions. The transitions for analysis of aesculin and IS were accordingly produced at *m/z* 341.2716 → *m/z* 179.1043 for aesculin and *m/z* 248.2035 → *m/z* 120.9130 for IS because these two transitions were specific and had the strongest intensities. Under the optimized conditions, good chromatographic separation and mass spectral signals were achieved in the assay of the plasma sample.

### 3.2. Matrix Effect and Extraction Recovery

Endogenous substances could interfere with aesculin determination due to the extreme low concentration of the compound in plasma. Using an appropriate internal standard is an important approach to reduce the matrix effects. In this study, tinidazole was used as internal standard. In Table 1, all the values (*A/B* × 100)% were between 94.2% and 106.4%, which means little matrix effect for aesculin and IS using the proposed method. The extraction recoveries of usnic acid from rat plasma were 97.6 ± 7.4, 95.8 ± 6.6, and 105.3 ± 4.1% at concentration levels of 40.0, 500.0.0, and 1200.0 ng/mL, respectively.

### 3.3. Method Validation

#### 3.3.1. Specificity

**Figure 1:**
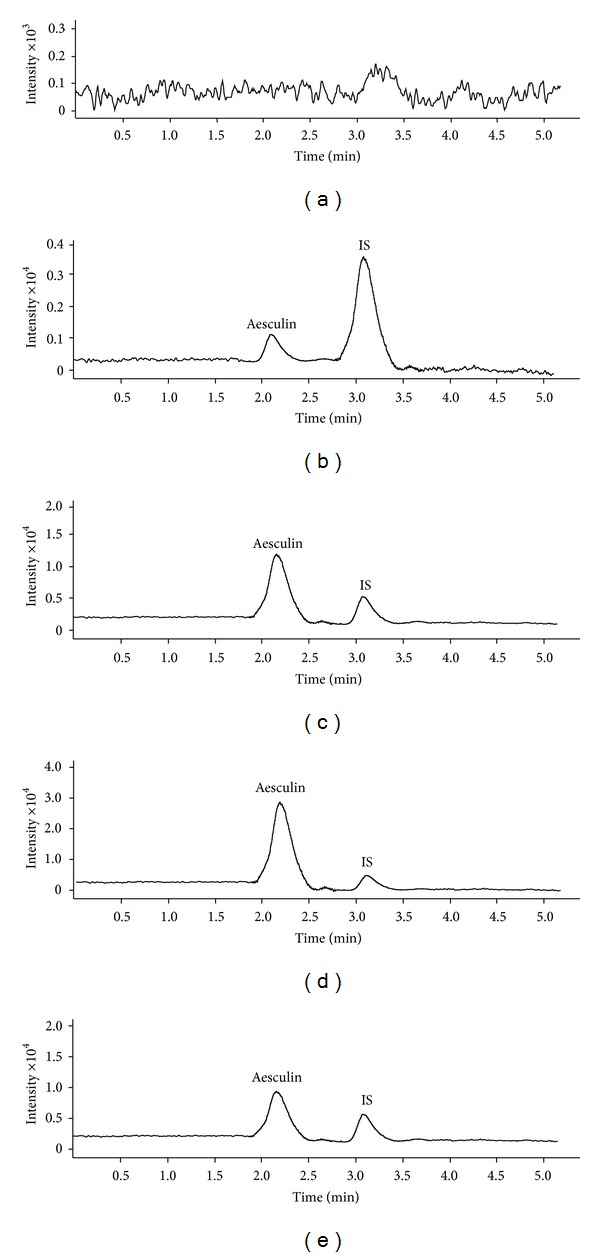
Representative extracted chromatograms for (a) blank plasma, (b) blank plasma containing 40.0 ng/mL aesculin and 10.0 ng/mL IS, (c) blank plasma containing 500.0 ng/mL aesculin and 10.0 ng/mL IS, (d) blank plasma containing 1200.0 ng/mL aesculin and 10.0 ng/mL IS, and (e) plasma sample collected at 0.17 after single oral dose of Cortex fraxini extract (5.0 mL/kg).

The base peak of each mass spectrum for aesculin and IS was observed from Q1 scans during the infusion of the neat solution in positive mode. Two precursor ions, *m/z* 341.2716 [M + H]^+^ for aesculin and *m/z* 248.3025 [M + H]^+^ for IS, were subjected to collision-induced dissociation (CID). The product ions were recorded as *m/z* 179.1043 [M-glu]^+^ and *m/z* 120.9130 [M-C_4_H_9_SO_2_]^+^, respectively. Mass transition patterns, *m/z* 341.2716 → 179.1043, and *m/z* 248.3025 → 120.9130, were selected to monitor aesculin and IS. Representative MS/MS extracted chromatograms of blank sample, low, medium, and high levels of QC samples plus plasma sample collected at 0.17 h after administration are shown in Figure 1. No endogenous peaks were found to be coeluted with the analytes, ensuring high specificity of the method. 

#### 3.3.2. Linearity and Sensitivity

Nine-point calibration curves were prepared ranging from 10.0 to 1500.0 ng/mL for aesculin. The regression parameters of slope, intercept, and correlation coefficient were calculated by 1/*x* weighted linear regression. Linearity was achieved with correlation coefficients greater than 0.9902 for all validation batches, shown in Table 2. The proposed method offered a limit of detection (LOD) of 2.0 ng/mL (*S/N* = 3) and a limit of quantitation (LOQ) of 8.0 ng/mL (*S/N* = 10), which is sensitive enough to investigate our pharmacokinetic behaviors of the compound. 

#### 3.3.3. Accuracy and Precision

The accuracy and the precision were analyzed by the QC samples at three concentrations. The assay accuracy and precision results are summarized in Table 3. The data obtained was within the acceptable limits to meet the guideline for bioanalytical methods (http://www.fda.gov). 

#### 3.3.4. Stability

The stability of aesculin in plasma tested in this work implied that no significant degradation occurred at room temperature for 7.0 h and at −20°C for 20 days. The plasma samples after three freeze and thaw cycles and the processed samples kept in the autosampler (4.0°C) for 48 h were stable. The stock solutions of aesculin and IS in methanol gave a good stability at 4°C for 15 days.

### 3.4. Comparison with Previous Methods

Chen et al. [16] have developed a high performance liquid chromatography method for determination of aesculin in rat plasma. In the report, the limit of detection was 24.0 ng/mL and the limit of quantification was evaluated to be 57.4 ng/mL. The method in this work is believed to be more sensitive than the previous method due to lower limits of detection and quantification. Another research by the same group [17] has developed a method using high performance liquid chromatography coupled to fluorescent detection for quantification of aesculin in rabbit plasma. The method gave a total run time of near 15 min, even using complicated sample preparation. Compared with the method, the assay in this work has high analyzing speed, simple sample preparation, and high selectivity from the selective ion patterns. All the above properties enable the application of the present method in pharmacokinetic study of aesculin.

### 3.5. Pharmacokinetics

**Figure 2:**
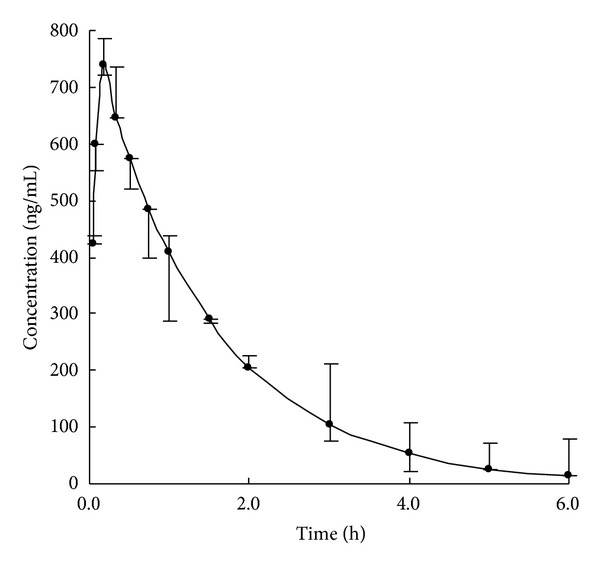
Mean plasma concentration-time profile of aesculin after administration of Cortex fraxini extract in rats (*n* = 6).

The method was successfully applied to the pharmacokinetic study of aesculin in rats. The mean plasma concentration-time profile of aesculin after administration is shown in Figure 2. The pharmacokinetic parameters of aesculin were calculated by the second version of an Excel software named Drug and Statistics (Shanghai, China). The pharmacokinetic process of aesculin was confirmed to be a one-compartment open model. The maximum plasma concentration (*C*_max⁡_) was calculated to be 739.5 ± 32.4 ng/mL with a value of 0.17 ± 0.02 h for the time to reach maximum plasma concentration (*t*_max⁡_). The half-life for absorption (*t*_1/2_) was 1.21 ± 0.03 h, providing the evidence that aesculin can exert its therapeutic effect quickly. The total exposures measured as AUC_(0–*∞*)_ (area under concentration-time curve) and AUC_(0–*t*)_ were calculated to be 2120 ± 152 and 1850 ± 167 ng/mL·h. The time points for plasma collection are accordingly believed to be acceptable due to the fact that ratio of AUC_(0–*∞*)_ versus AUC_(0–*t*)_ is lower than 120%. Accordingly, the proposed method is believed to be suitable to the determining aesculin in biological fluids and will probably be an alternative for pharmacokinetic study on aesculin.

## 4. Conclusion

A sensitive, selective, and rapid HPLC-QTOF-MS/MS assay for monitoring aesculin in rat plasma was developed. The method proves to be capable of reducing ion suppression and offering superior sensitivity with an LOQ of 10.0 ng/mL, satisfactory selectivity, and short run time less than 4.0 min. The method has been successfully applied in a pharmacokinetic study of aesculin in rat, providing an alternative for clinical determination of the compound.

## Figures and Tables

**Table 1:** Matrix effects and extraction recovery of aesculin and the internal standard in rat plasma.

Analytes	Concentration (ng/mL)	Matrix effects (%, *n* = 6)	CV (%)	Extraction recovery (%, *n* = 5)	CV (%)
Aesculin	40.0	98.1 ± 5.6	5.7	97.6 ± 7.4	7.6
	500.0	106.4 ± 3.8	3.6	95.8 ± 6.6	6.9
	1200.0	94.2 ± 6.9	7.3	105.3 ± 4.1	3.9

IS	10.0	96.2 ± 3.3	3.4	97.5 ± 5.8	5.9
	10.0	100.6 ± 5.2	5.2	104.7 ± 4.8	4.6
	10.0	104.2 ± 4.1	3.9	98.2 ± 3.9	4.0

**Table 2:** Linearity for assay of aesculin in rat plasma.

Analytical batch	Slope	Intercept	Regression equation	Correlation coefficient
1	0.0365	0.0332	*y* = 0.0365*x* + 0.0332	*r* = 0.9902
2	0.0384	0.0341	*y* = 0.0384*x* + 0.0341	*r* = 0.9915
3	0.0352	0.0337	*y* = 0.0352*x* + 0.0337	*r* = 0.9946
4	0.0379	0.0354	*y* = 0.0379*x* + 0.0354	*r* = 0.9952
5	0.0369	0.0327	*y* = 0.0369*x* + 0.0327	*r* = 0.9929

**Table 3:** Intraday (*n* = 5) and interday (*n* = 5) precision and accuracy for assay of aesculin in rat plasma.

Concentration (ng/mL)	Intraday (*n* = 5)			Interday (*n* = 5)		
	Mean ± SD (ng/mL)	RSD (%)	Accuracy (%)	Mean ± SD (ng/mL)	RSD (%)	Accuracy (%)
40.0	40.2 ± 1.4	3.5	100.5	38.5 ± 1.2	3.1	96.3
500.0	487.5 ± 15.2	3.1	97.5	490.7 ± 11.6	2.4	98.1
1200.0	1226.0 ± 86.3	7.0	102.2	1217.0 ± 75.8	6.2	101.4
